# Treatment and follow up of children with chronic hepatitis C in Albania

**DOI:** 10.1186/1743-422X-9-17

**Published:** 2012-01-13

**Authors:** Virtut Velmishi, Ermira Dervishi, Paskal Cullufi, Donjeta Bali, Vjollca Durro

**Affiliations:** 1 Service of Pediatric Gastrohepatology, University Hospital Center "Mother Theresa", Rruga "Dibres" No. 372, Tirana, Albania; 2 Service of Pediatric Oncohematology, University Hospital Center "Mother Theresa", Rruga "Dibres" No. 372, Tirana, Albania; 3 Hospital Planning Directory, Ministry of Health, Bulevardi " Bajram Curri", no 1, Tirana, Albania

**Keywords:** Chronic Hepatitis C = CHC, HCV = Hepatitis C Virus, SVR = Sustained Viral Response, ETVR = End Treatment Viral Response

## Abstract

**Background:**

Treatment of Hepatitis C in children has a better outcome than in adults, and for this reason the treatment had different views. However, in pediatric age hepatitis C is seen to have an evolution towards chronicity. Today is a normal option to treat chronic hepatitis C as early as possible according to certain criteria. The aim of this study is to show the results of treatment with interferon and ribavirin and the follow-up of children diagnosed with chronic hepatitis C in our service.

**Patients and methods:**

This is a prospective study which has included children 3 up to 15 years old (13 boys and 4 girls) diagnosed with chronic hepatitis C. All patients underwent a certain protocol, including liver biopsy prior to treatment. Treatment consisted in use for 48 weeks of INF α-2b, 3 MIU/m2 three times a week s/c and ribavirin 15 mg/kg orally divided bid. Two patients were treated with PEGINF α-2b with dose 1.5 mcg/kg once a week s/c and ribavirin 15 mg/kg. After the treatment all patients have stayed under our control for an average period of 24 weeks.

**Results:**

At the end of the treatment we detected a patient with HCV-RNA positive. End Treatment Viral Response was 94%. Six months later we found three patients who showed relapse of disease. Sustained Viral Response was approximately 83%

**Conclusion:**

The combination therapy of interferon with Ribavirin in treatment of children with chronic hepatitis C provides a higher SVR when treatment is initiated at the earliest stages of hepatic changes. Side effects of therapy are insignificant in comparison with results obtained

## Background

The combined treatment of Interferon and ribavirin is currently approved for treatment of chronic hepatitis C in children. Today, Peg interferon is found more convenient because of the weekly administration (once a week versus three times per week of conventional INF) as a result of its long plasmatic half-life which is provided by covalently linking a polyethylene glycol moiety to the INF. There are not many studies on the effect of PEG-INF in children and adolescents [1] However they show that the efficiency of treatment is better in forms with minimal histopathological changes, with low levels of viral load, and with genotype other than genotype 1 [2-4]. An international study has observed pharmacokinetics, efficacy and safety of PEG-INF-α-2b 60 μg/m2/week combined with ribavirin 15 mg/kg/weight in 107 children. Another study is conducted in North America where the relative efficacy of the combination of PEG-INF α-2a and ribavirin (55 children) is compared with PEG-INF-α-2a as monotherapy (59 children). Over time and in parallel with the studies in adults, experience has been gained in children with INF-α monotherapy [5-9], the combined use of INF-α and ribavirin [10,11], PEG-INF monotherapy [12] and finally the combination of PEG-INF with ribavirin, which is approved by the US Food and Drug Administration (FDA) in December 2008 for use in children over 3 years old.

The aim of our study is to show the results of treatment with interferon and ribavirin and the follow-up of children diagnosed with chronic hepatitis C in our service. These patients have received multitransfusions as a part of their therapy during treatment of their primary illness. The prevalence of HCV is blood donor population in Albania is 0.7% [13].

## Patients and methods

Since November 2007 until March 2010, in our service of Pediatric Gastrohepatology, we have treated 17 children diagnosed with CHC. Diagnosis of these children is performed through the Anti-HCV confirmed later with HCV-RNA. We haven't had any case of HCV associated with hepatitis B, D, HIV or other pathology which would severely damage liver function. The most part of the patients, 14 of them came from the service of Oncohematology where they were treated for their illnesses. All these children were in full remission of their basic disease or were considered cured and had at least 12 months without treatment with cytostatics or immunosuppressive agents. All the patients who came from oncohematology service have received multi-transfusions. The age of the children was from 3 to 15 years old. We had 13 boys and 4 girls. Two cases showed hepatitis C with unknown transmission route and a child is diagnosed with Gaucher disease treated with replacement enzyme-therapy. We didn't find any patient with vertical transmission of the disease.

## Method

Treatment which was carried out according to a protocol where is combined the use of interferon α2b (2 children were treated with PEG-INF-α-2b) and ribavirin, with a dose of 3MIU/m2 s/c three times a week for INF-α-2b and 1.5mcg/kg s/c once a week for PEG-INF-α-2b. The dosage of ribavirin has been 15 mg/kg/d divided bid. A protocol was composed to follow up patients during the treatment as shown in Table 1.

**Table 1:** The protocol of children during one year of treatment

Before beginning the treatment	total blood count, liver function test, GGT(gamma glutamyl transferase), albumine, protein electrophoresis, total protein, protrombine time, HCV RNA *, genotype, liver biopsy
**After 1month**	total blood count, liver function test, protrombine time, GGT, albumine, total protein, protein electrophoresis, sedimentation rate

**After 3 months**	as the first month plus HCV RNA

**After 6 months**	as the third month

**After 9 months**	as the first month

* Due to the high cost HCV RNA and genotype was not performed under this Protocol. HCV RNA is accomplished before and at the end of the treatment and approximately 6 months after treatment to assess SVR. Genotype is carried out for only three patients, who resulted 1b

### Ethical approval

This study is carried out in compliance with the Helsinki Declaration known by Albanian Committee on Bioethics (Rr. Reshit Petrela No 27, Tirana-Albania). The parents of all children included in this study provided their consent

## Results

Out of 17 children, 14 were first treated in pediatric service of oncohematology with blood transfusions or its by-products. This fact explain the main reason of HCV transmission thorough transfusion. Two other children had an unknown transmission of hepatitis C and a patient with Gaucher disease. To each patient was performed liver biopsy prior to treatment as show in Tables 1 and 2. Other biochemical data (liver tests, aminotranferases, PT, protein electrophoresis etc.) are not presented in the summary table because the most part of patients have had at the beginning of therapy normal values of aminotransferases or slightly increased (1-2 times) without hyperbilirubinemia.

**Table 2:** Summary Chart of patients

Patients	Age	Sex	Diagnosis	HCV-RNA	Liver Biopsy	Treatment	Side effects	HCV-RNA	Genotype
1	11 y	M	Non-Hodgking lymphoma	1.5 × 10^6 ^copies /ml	Metavir**A1F1	INFα2b***Ribavirin	Leucopenia	Not detected	

2	5 y	M	Non-Hodgking lymphoma	1.8 × 10^6 ^copies /ml	Metavir A2F2	INFα2b***Ribavirin	None	Detected	1B

3	5 y	M	Testicular teratocarcinoma	761,000 copies/ml	Metavir A2F0	INFα2b***Ribavirin	None	Not detected	

4	6 y	F	Meduloblastoma	6.3 × 10^4 ^copies/ml	Metavir A1F2	INFα2b***Ribavirin	Leucopenia headache	Not detected	

5	12 y	M	Testicular seminoma	9.2 × 10^4 ^copies/ml	Metavir A2F1	INFα2b***Ribavirin	Fever	Not detected	

6	11 Y	M	Leukemia	3,600 copies/ml	Metavir A1F1	INFα2b***Ribavirin	None	Not detected	

7	10 y	F	Non-Hodgkin lymphoma	1.1 × 10^6 ^copies /ml	Metavir A3F2	INFα2b***Ribavirin	None	Not detected	1B

8	6 y	F	Leukemia	1.8 × 10^6 ^copies /ml	Metavir A2F1	INFα2b***Ribavirin	None	Not detected	

9	3 y	M	Hemolytic anemia	1.5 x10^6 ^copies /ml	Metavir A1F2	INFα2b***Ribavirin	None	Not detected	

10	8 y	M	Gaucher	6.8 × 10^5 ^copies/ml	Metavir A2F1	INFα2b***Ribavirin	None	Not detected	

11	8 y	M	Leukemia	1.6 × 10^6 ^copies /ml	Metavir A2F1	INFα2b***Ribavirin	Myalgia	Not detected	1B

12	11 y	M	Unknown	1.8 × 10^6 ^copies /ml	Metavir A1F2	INFα2b***Ribavirin	Headache	Not detected	

13	10 y	M	Leukemia	56,700 copies /ml	Metavir A2F2	INFα2b***Ribavirin	Fever	Not detected	

14	15 y	F	Faringeal Ewing -sarcoma	9.3 × 10^5^copie/ ml	Metavir A1F0	PEGINFα2b *** Ribavirin	None	Not detected	

15	10 y	M	Leukemia	1.8 × 10^6 ^copies /ml	Metavir A2F1	INFα2b***Ribavirin	None	Not detected	

16	10 y	M	Unknown	1.5 x10^6 ^copies /ml	Metavir A2F1	INFα2b***Ribavirin	None	Not detected	

17	8 Y	M	Infantile fibrosis	2.5 x10^6 ^copies /ml	Metavir A2F2	PEGINFα2b *** Ribavirin	Fever, Cramps, Vomiting	Not detected	

*HCV-RNA are shown at the beginning and at the end of treatment. At the end we had only one patient with positive HCV RNA. ETVR = 94%. To each patient is performed an other HCV RNA of control (6 months after treatment to determine SVR) which has resulted positive for three patients. SVR was approximately 83%

** METAVIR is a histopathological score which expresses the rank of inflammation (A) and hepatic fibrosis (F) in cases of hepatitis C, etc.. Missing -A0, A1-minimum, A2-moderate; A3-severe; F0-no fibrosis; F1-portal fibrosis non-septal l; F2-portal fibrosis with few septal fibrosis; F3- septal fibrosis without cirrhosis, F4-cirrhosis

***Multi-transfusion patient (patient who has received more than 3 units of blood products

All our patients have had a moderate number of copies of HCV RNA (only one patient with over 2 million copies per ml). The prevalent histopathological pattern is chronic hepatitis with minimal or moderate lesions, but there isn't any case with cirrhosis (F4) or septal fibrosis (F3). The most frequent hematological side effects were leucopenia which has been moderated by allowing us to continue treatment. Another side effect, not very rare, was fever and headache which were present at the beginning of the treatment (Table 2). At the end of treatment, only one patient was detected with HCV RNA positive. End Treatment Viral Response(ETVR)^1 ^was 94%. Six months after the treatment we found only three patients with HCV RNA positive. Sustained Viral Response(SVR)^2 ^was approximately 83%

## Discussion

Chronic hepatitis C in children and teens is usually silent (hepatitis c is known as "silent killer") [14]. Hystopathological changes in most of cases are minimal showing mild degrees of inflammation and fibrotic changes. A liver biopsy is recommended before beginning the treatment. Some serious studies performed in children with chronic hepatitis C in most of cases have shown few histological lesions and the evaluation by biopsy has been controversy, but still today remains the only method to evaluate the severe cases [15-17] Only 1.3% (one in 80 children) can achieve to cirrhosis [18,19]. Theoretically cirrhosis could be developed after a mean time of 28 years [20,21]. The progress of fibrosis is depended on the duration of infection when we don't have other risk factors that damage liver function. Some other authors did not associate hepatic injury with the duration of infection [22].

It have to be noted that we cannot predict the age when this pathology can be more aggressive. Nevertheless children should spend a few decades being ill, until the risk of resistance to treatment appears. A careful treatment of patients with chronic infection, early in their life is preferable to prevent the progression of the disease [23-27]. In our study we achieved a SVR approximately 83%, while in other serious studies they reached values around 53% SVR for genotype 1, 93% for genotype 2 and 3 and 80% for genotype 4. The relative high result can be explained as below mentioned:

First of all, we were unable to define genotype for all patients, but other studies conducted in our country from Public Health Institute(unpublished data), indicate that genotype 1 is more frequently. For this reason it was decided to extend treatment up to 48 weeks.

It is thought that if we had predominantly "mild" genotype except G1 means that a higher SVR is expected. Secondly, we note that in our cases viral load was relatively low. Today is known as "baseline" level of viral load ≤600,000 UI/ml or 2,000,000 copies/ml [28]. We had only one patient with over 2,000,000 copies/ml (Table 2), which means a higher SVR.

Thirdly, the high result achieved can be explained from minimal histopathology changes encountered in 17 patients. According to the METAVIR evaluation we have not had any patient in F3 level (septal fibrosis with few septa) or F4 (cirrhosis).

Above all, in our study, the use of INF combined with ribavirin has been very well tolerable by all patients. Among the most frequent side effects were fever, headache, etc. known as "flu like symptoms". Hematological side effect was dominated by a moderate leucopenia so we weren't constrained to interrupt the treatment. We hadn't any child with anemia induced by ribavirin, so we administrated a full dose in all patients. We were unable to bring out an accurate conclusion about the impact of INF on the impairment of growth velocity, because we had a limited number of children involved in this study. Today there are serious studies that assess the growth of children during the treatment with INF and 5 years later without treatment [29,30].

During the treatment we didn't observe changes in school performance or psychological disorders. We hadn't any child with thyroid dysfunction [31,32]. Finally, the combined therapy of INF with Ribavirin in children and adolescents with chronic hepatitis C, in the earliest stage and with low viral load, is associated with higher SVR. Our data have also shown a very good tolerance of this combined therapy in pediatric ages. The beginning of treatment is preferable before the period of adolescence.

## Endnotes

^1^"End Treatment Viral Response" is defined as undetectable serum HCV RNA in the end of treatment

^2^"Sustained Viral Response" is defined as undetectable serum HCV RNA 6 months after treatment

## Competing interests

The authors declare that they have no competing interests.

## Authors' contributions

VV drafted the manuscript. ED performed the statistical analysis. PC conceived of the study, and participated in its design and coordination and helped to draft the manuscript. DB participated in the sequence alignment and carry out the corresponding reference. VD was involved in drafting and revising the manuscript. All authors read and approved the manuscript
